# Efficacy of Saffron as an Adjunct to Methylphenidate in Treating ADHD in Children Aged 6–12: A Randomized Clinical Trial

**DOI:** 10.1002/brb3.71822

**Published:** 2026-10-19

**Authors:** Asieh Hosseinpourfardi, Seyyed Mohammad Saeid Sahaf, Fahimeh Afzaljavan, Atefeh Soltanifar, Fatemeh Moharreri

**Affiliations:** ^1^ Psychiatry and Behavioral Sciences Research Center Mashhad University of Medical Sciences Mashhad Iran; ^2^ Department of Neuroscience, Faculty of Medicine Mashhad University of Medical Sciences Mashhad Iran; ^3^ Neuroscience Research Center Mashhad University of Medical Sciences Mashhad Iran; ^4^ Student Research Committee, Faculty of Medicine Mashhad University of Medical Sciences Mashhad Iran

**Keywords:** *Crocus sativus*, crocin, impulsivity, inattention, methylphenidate

## Abstract

**Introduction:**

Attention‐deficit/hyperactivity disorder (ADHD) is a common childhood disorder often managed with stimulant medications such as methylphenidate. Around 30% of patients with ADHD do not respond to treatment or cannot tolerate its side effects, which necessitates the consideration of alternative options. This study evaluated the effectiveness of adding the saffron extract to methylphenidate on ADHD symptoms and working memory in children aged 6–12.

**Method:**

In a randomized clinical trial, 58 children diagnosed with ADHD were randomized into two groups: a methylphenidate (0.5–1 mg/kg/day, divided into 2–3 doses)/saffron (20–30 mg/day based on weight: 20 mg for <30 kg, 30 mg for >30 kg) group and a control group receiving only methylphenidate (0.5–1 mg/kg/day, divided into 2–3 doses) for six weeks. The severity of ADHD was evaluated using subjective (Conners' Parent Rating Scale‐revised) and objective (Corsi block‐span task and continuous performance test) tests at the start and end (in the sixth week) of the study.

**Results:**

22 participants in the methylphenidate/saffron group and 21 in the control group completed the study. The changing trend of total ADHD scores (*p* = 0.002, Cohen's d = 0.85), hyperactivity score (*p* = 0.024, d = 0.62), impulsivity score (*p* = 0.031, d = 0.58), and attention score (*p* = 0.001, d = 0.92) was significantly different between groups based on subjective parent reports. However, no significant differences were found in impulsivity, inattention, or sustained attention on the CPT, though reaction time improved significantly (*p* = 0.023, d = 0.45). Scores on the Corsi Block Span Task indicated no significant changes in working memory between groups. The combination treatment was well‐tolerated, with no reports of severe adverse effects.

**Conclusion:**

The findings suggest that the combination of saffron with methylphenidate may enhance subjective reports of ADHD symptoms, particularly hyperactivity and impulsivity; however, objective cognitive measures showed limited changes. Given the favorable safety profile of saffron and study limitations, this supports its potential role as a complementary option, pending confirmation in placebo‐controlled trials with larger samples and long‐term evaluations.

## Graphical Abstract

This study demonstrates that adding saffron extract to methylphenidate significantly improves ADHD symptoms, particularly reducing hyperactivity and impulsivity, without severe side effects. These findings suggest saffron may serve as a beneficial complementary treatment for children with ADHD.

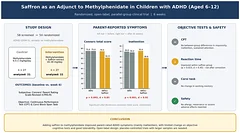

## 1 Introduction

Attention‐deficit/hyperactivity disorder (ADHD) is a chronic neurodevelopmental disorder characterized by persistent inattention and/or hyperactivity‐impulsivity behavior in children. It is a prevalent pediatric mental health condition, with a global estimated prevalence of 7.6% (95% CI 6.1%–9.4%) in children aged 3 to 12 years and 5.6% (95% CI 4.8%–7%) among teenagers aged 12 to 18, higher in boys (Salari et al. 2023). ADHD significantly affects daily functioning, peer relationships, education, and well‐being, straining individuals, families, and societal structures (Canals Sans, Morales Hidalgo, Roigé Castellví, Voltas Moreso, and Hernández Martínez 2021; A. K. Mueller, Fuermaier, Koerts, and Tucha 2012).

According to the Diagnostic and Statistical Manual of Mental Disorders, Fifth Edition (DSM‐5), diagnosing ADHD requires identifying developmentally inappropriate inattention and/or hyperactivity‐impulsivity that appear before age 12 and impair functioning across various contexts (Regier, Kuhl, and Kupfer 2013). Conventional treatment involves behavioral and pharmacological strategies, with stimulants, such as methylphenidate, central to management (Nazarova et al. 2022). Despite their efficacy, 20%–40% show resistance or side effects, including growth stunting (Chen et al. 2019), necessitating alternatives.

The past two decades have seen a rise in the use of Traditional, Complementary, and Alternative Medicines (TCAMs), with 24%—71.3% of the global population reporting recent use (Asgharian et al. 2022; Küpeli Akkol et al. 2021; Lee et al. 2022). Herbal Medicine (HM) is prominent, with the WHO estimating that 70%–90% of individuals in rural developing countries rely on HM for various healthcare needs (Shirwaikar, Govindarajan, and Rawat 2013). Given concerns about conventional pharmacological treatments, including long‐term adverse effects and challenges in access to behavioral therapies, many parents are turning to alternative options (Handelman and Sumiya 2022).

Excitingly, a recent randomized clinical trial (RCT) suggests saffron may offer a compelling alternative, demonstrating efficacy comparable to methylphenidate for ADHD symptom reduction (Baziar et al. 2019). However, that study lacked a placebo control, raising questions about placebo effects or regression to the mean, and had a small sample size with a short duration, limiting generalizability. A recent systematic review summarized saffron's effects on ADHD but noted limitations like small samples and short durations, justifying our focus on adjunctive use with objective measures (Seyedi‐Sahebari et al. 2024). Saffron, derived from *Crocus sativus L*., has been traditionally used as a culinary spice and for diverse medicinal purposes (Cardone et al. 2020). Traditionally, saffron has been employed as a food seasoning and has medicinal uses within traditional Iranian medicine for various conditions (Mollazadeh et al. 2015). Its bioactive compounds, particularly carotenoids, exhibit notable antioxidant and anti‐inflammatory properties, further suggesting its therapeutic potential for neuropsychiatric conditions (Cerdá‐Bernad, Valero‐Cases, Pastor, and Frutos 2022). Saffron's crocin and safranal may modulate dopamine and serotonin systems, which are implicated in ADHD etiology involving imbalances in dopaminergic and noradrenergic neurotransmission (Genro, Kieling, Rohde, and Hutz 2010), offering neuroprotective effects against ADHD‐related neurotransmission imbalances (Bian, Zhao, and Lee 2020). The rationale for combining with methylphenidate is potential synergy: saffron's neuroprotective effects may enhance stimulant efficacy while mitigating side effects. However, while initial findings are promising, comprehensive clinical trials are imperative to validate saffron's role in ADHD management and its effects on working memory and sustained attention among children aged 6–12.

## 2 Methods

### 2.1 Ethics

All experimental procedures adhered to ethical standards established by the institutional and national research committees, which were in line with the 1964 Helsinki Declaration and its later amendments. The study protocol received approval from the Ethics Committee of “details omitted for double‐anonymized peer review” (Ethics Number: 1402.386). The study is registered in the Iranian Clinical Trial Registration Center (Registration Number: “details omitted for double‐anonymized peer review”). Before participation, parents (either father or mother) were thoroughly informed of the intervention procedures, potential side effects, and benefits and provided written informed consent.

### 2.2 Study Design

This study was designed as a randomized, parallel‐group, superior clinical trial to evaluate the impact of saffron extract supplementation on ADHD symptoms and working memory in children aged 6 to 12.

### 2.3 Study Population

The study targeted children aged 6 to 12 years diagnosed with ADHD according to DSM‐5 criteria, who were referred to “details omitted for double‐anonymized peer review.” Demographic information, such as age and gender, was recorded for all participants.

### 2.4 Inclusion and Exclusion Criteria

Inclusion criteria were children aged 6 to 12, confirmed diagnosis of ADHD by a child and adolescent psychiatry specialist based on DSM‐5 criteria, parents provided written informed consent for participation, and normal IQ levels (assessed via a standardized clinical evaluation).

Exclusion criteria were non‐cooperation from parents at any stage of the study, presence of any other significant psychiatric disorders or systemic diseases requiring medication, substance dependence identified in the participant, and inability to complete detailed examinations.

### 2.5 Randomization and Blinding

Simple randomization was performed using a computer‐generated randomization method to allocate participants into intervention and control groups. The study was not blinded, as the control group received no placebo; however, the evaluator and data analysts were blinded to group assignments to mitigate bias in outcome assessment.

### 2.6 Intervention

The intervention comprised saffron capsules containing 10 mg of dry saffron extract, which could be dissolved in beverages or food for participants with swallowing difficulties. The dosage regimen included one capsule before breakfast and one capsule before dinner for children weighing under 30 kg, and one capsule before breakfast, one capsule before lunch, and one capsule before dinner for children weighing over 30 kg. Dosing was based on prior RCTs (Baziar et al. 2019; Khaksarian et al. 2021; Pazoki et al. 2022), adjusted for weight to ensure safety.

The saffron preparation used was a branded crocin capsule containing 10 mg of dry saffron extract per capsule, derived from the stigma of *Crocus sativus L*. (the plant part traditionally used for medicinal purposes). The capsules were produced by a licensed pharmaceutical manufacturer in Iran. The extraction method involved water‐based extraction of the dried stigmas to obtain a concentrated dry extract, standardized to contain key active molecules, including crocin (the primary carotenoid responsible for neuroprotective effects) and safranal (contributing to aroma and potential GABAergic activity).

### 2.7 Study Procedure

Fifty‐eight children aged 6 to 12 who attended the child and adolescent psychiatry clinic at “details omitted for double‐anonymized peer review” were screened for inclusion criteria. The evaluation involved an organized psychiatric interview conducted by a qualified child and adolescent psychiatrist, assessing ADHD symptoms as per DSM‐5 criteria and confirming they had been on methylphenidate treatment for at least three months. Four subjects were excluded due to the following reasons: lack of parental consent (1) and diagnosis of other psychiatric disorders (3). Consequently, 54 participants were enrolled in the study.

After explaining the study objectives, obtaining informed consent from the parents, and using the computer randomization method, participants were randomly assigned to intervention (n = 27) or control (n = 27) groups.

Before the intervention, ADHD symptoms were evaluated using the Conners' Parent Rating Scale‐Revised (CPRS‐R). Attention deficits were assessed using the continuous performance test (CPT) while working memory was evaluated using the Corsi Block Span Task (CBST). Assessments (CPRS‐R, CPT, CBST) were conducted in the morning (8–11 AM), approximately 1–2 h post‐morning methylphenidate dose, to capture peak medication effects. Throughout the intervention period, all participants continued treatment with methylphenidate at a dosage of 0.5–1 mg/kg/day, divided into 2–3 doses per day. In addition to methylphenidate treatment, children in the intervention group received saffron capsules for six weeks, as specified above.

The intervention and control group patients were evaluated again after six weeks using Conners' Parent Rating Scale‐revised, CBST, and CPT.

### 2.8 Outcome Assessment

Primary outcomes included changes in scores of impulsivity, attention deficits, and hyperactivity in children with ADHD, evaluated using the Conners' Parent Rating Scale‐Revised. Secondary outcomes were assessed through the CBST and CPT scores. Measures were selected to capture both subjective (parental) and objective (cognitive) aspects, aligning with ADHD guidelines.

### 2.9 Instruments

#### 2.9.1 Conners' Parent Rating Scale‐Revised

The CPRS‐R, developed by Conners et al. in 1997, consists of 27 items assessing behavioral and emotional problems in children. The scale includes four cognitive subscales: inattention (6 items), hyperactivity (6 items), impulsivity (6 items), and the Conners Index (9 items). Parents rate the frequency and severity of each behavior on a 4‐point Likert scale (1 = Not True at All to 4 = Very Much True), yielding a total score ranging from 27 to 108. In a study by Shaheaian et al. in Shiraz, Iran, the CPRS‐R was standardized for primary school children, achieving a test‐retest reliability coefficient of 0.58 and an internal consistency (Cronbach's alpha) of 0.73. Validity assessments indicated strong correlations between subscales and the overall score, with coefficients ranging from 0.76 to 0.90 (Shahaeian et al. 2007).

#### 2.9.2 Continuous Performance Test

The CPT is a widely recognized assessment tool for evaluating sustained attention. This test measures an individual's ability to maintain focus on a designated task while inhibiting responses to distracting stimuli. During the CPT, participants view a rapid succession of visual stimuli and are instructed to respond only to specific target stimuli while withholding responses to non‐targets. Key performance metrics include sustained attention, reaction time, and the frequency of commission errors (incorrect responses to non‐targets) and omission errors (failure to respond to targets). In our study, participants focused on a central box displayed on a 15.6‐inch screen, where various stimuli (e.g., gloves, sun, stars) appeared. Participants were instructed to press the spacebar in response to the appearance of a moon. Commission errors were interpreted as indicators of impulsivity, omission errors as indicators of inattention, and the total number of correct responses as reflective of sustained attention, alongside recorded reaction times (Khodadadi et al. 2014; Piper et al. 2016).

#### 2.9.3 Corsi Block‐Span Task

The CBST is a well‐established cognitive assessment for measuring working memory. This task involves nine blocks arranged in a specific order, where participants must observe and memorize the sequence of presented blocks. Subsequently, participants are asked to reproduce the sequence in reverse order (i.e., reporting the last presented block first). The test begins with a span of two consecutive blocks and increases in difficulty with longer sequences. The assessment concludes after two consecutive failures in two attempts. The final score is calculated by multiplying the span of the last successfully recalled sequence by the total number of correct attempts. The CBST is recognized for its effectiveness in evaluating working memory components. In this study, we focused on the backward span, interpreting the total number of correct responses as the Working Memory (WM) Score and the longest sequence correctly recalled as the WM Span (Kessels et al. 2000; S. T. Mueller and Piper 2014).

### 2.10 Side Effect Evaluation

During the trial, participants were closely monitored for unexpected symptoms or adverse effects, including mood changes, sleep disturbances, and gastrointestinal issues. Adverse events were monitored weekly via parent phone interviews and clinic visits, using a standardized checklist. While saffron is generally considered safe at recommended doses, any participant exhibiting intolerance or rare side effects was promptly withdrawn from the intervention. Symptomatic treatment was initiated in such cases, and all adverse effects were documented.

### 2.11 Sample Size Calculation

Based on a previous study, the average scores of the Attention‐Deficit/Hyperactivity Disorder Rating Scale‐IV (ADHD‐RS‐IV) for parents in the methylphenidate group and the methylphenidate plus saffron group at the end of the trial were 27.15 ± 9.46 and 19.07 ± 6.90, respectively (Khaksarian et al. 2021). Using an alpha level of 0.05 and a beta level of 0.10, the sample size was calculated to be 24 participants per group using G*Power 3.1.9.4. To account for a potential dropout rate of 10%, we aimed to enroll 27 participants in each group.

### 2.12 Statistical Analysis

Data analysis was conducted using SPSS v 23. Descriptive statistics, including means ± standard deviations and frequencies, were employed to summarize the characteristics of the study population. Normality was assessed via the Shapiro‐Wilk test. Independent t‐tests or their non‐parametric equivalents were utilized to compare quantitative variables between the two groups, while chi‐square tests were used for qualitative variables. Effect sizes were calculated using Cohen's d. Statistical analyses were conducted on a per‐protocol basis, including only participants who completed the study and adhered to the protocol. Intention‐to‐treat (ITT) analysis was not performed due to the lack of post‐baseline data for dropouts. A *p*‐value of <0.05 was considered statistically significant. To account for multiple comparisons and reduce the risk of Type I errors, the Bonferroni correction was applied separately to the primary outcomes (changing trends in Conners' Parent Rating Scale‐Revised subscales: total score, hyperactivity, impulsivity, and inattention; m = 4 tests, adjusted α = 0.05/4 = 0.0125) and secondary outcomes. For secondary outcomes, correction was applied within each instrument: CPT metrics (changing trends in impulsivity, inattention, sustained attention, and reaction time; m = 4 tests, adjusted α = 0.05/4 = 0.0125) and CBST metrics (changing trends in working memory span and working memory score; m = 2 tests, adjusted α = 0.05/2 = 0.025). Note that the statistical population for the Conners' Parent Rating Scale‐Revised consists of parent‐reported data on children's symptoms, while for the CPT and CBST, it consists of direct assessments on the children.

## 3 Results

The study flow is reported based on the CONSORT guideline in Figure 1. Among the 27 children included in the methylphenidate/saffron group, five subjects discontinued participation for various reasons: two due to lack of consent to continue, two due to irregular consumption of the adjuvant medication, and one for failing to return to complete the final assessment. Similarly, six participants in the control group withdrew from the study: one required additional medications, three did not return to complete the final assessment, and two withdrew consent to continue. Consequently, data from 22 participants in the methylphenidate/saffron group and 21 participants in the control group were analyzed.

**FIGURE 1:**
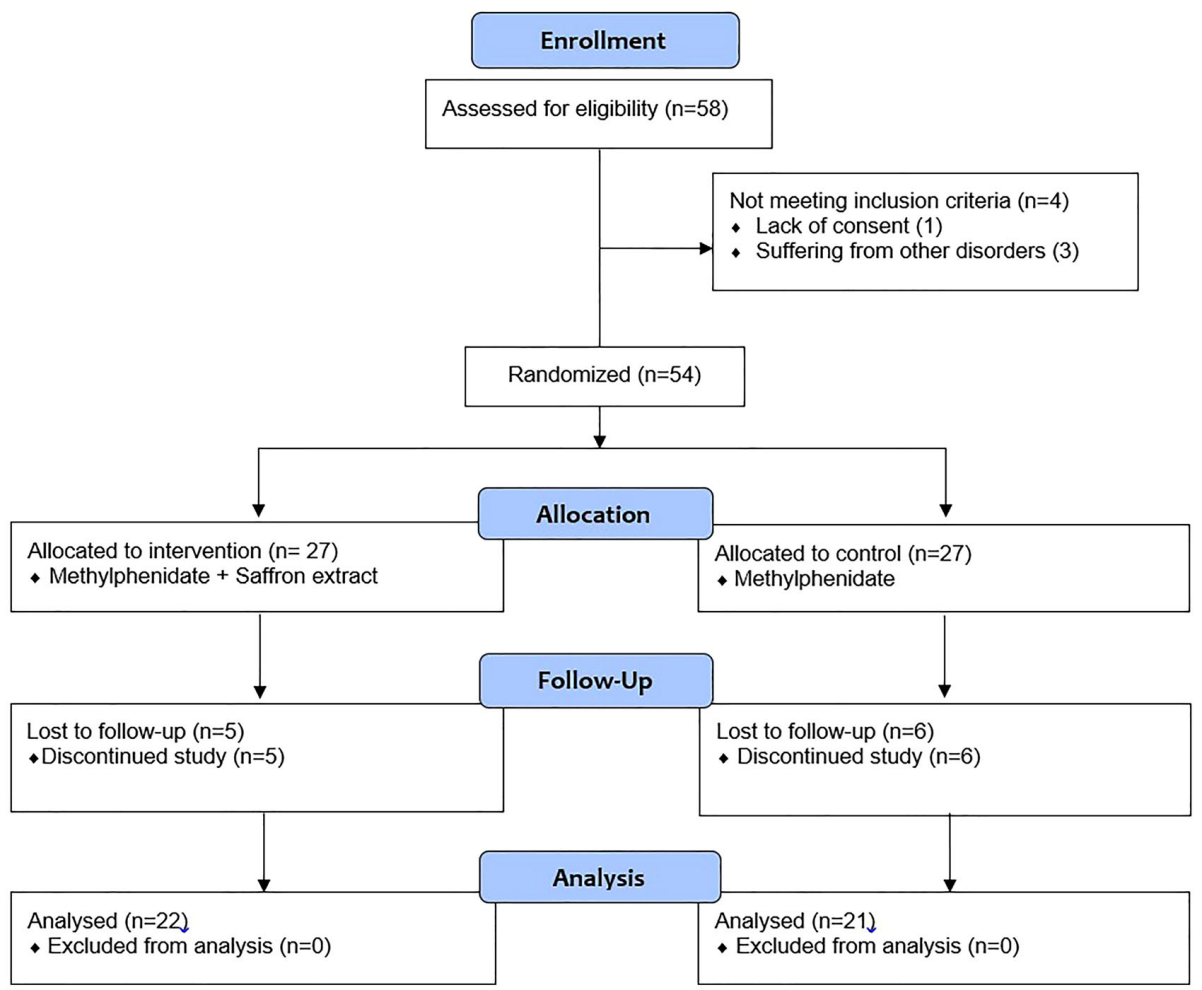
CONSORT diagram of the study population.

Table 1 presents demographic data. The mean age of participants was 9.00 ± 2.05 years in the control group and 8.91 ± 2.04 years in the intervention group (*p* = 0.885). The gender distribution was comparable between the groups (*p* = 0.537), with the control group comprising 12 boys (57.1%) and 9 girls (42.9%), compared to 15 boys (68.2%) and 7 girls (31.8%) in the intervention group.

**TABLE 1:** The characteristics of the study population.

Characteristics		Methylphenidate	Methylphenidate/saffron	*p*‐value
**Age**		9.00 ± 2.05	8.91 ± 2.04	0.885
**Sex**	Male	12 (57.1%)	15 (68.2%)	0.537
	Female	9 (42.9%)	7 (31.8%)	

As shown in Table 2, ADHD symptoms reported by parents were compared between the intervention and control groups, as well as within each group across different study time points. While baseline Conners‐related scores were similar between the groups, significant differences were observed post‐intervention: the total score (*p* = 0.009), hyperactivity score (*p* = 0.019), and impulsivity score (p = 0.006) were all significantly lower in the methylphenidate/saffron group compared to the control group. Additionally, there was a significant improvement in all Conners scores from the start to the end of the study within the intervention group. The changing trends in the Conners’ total score (*p* = 0.002), hyperactivity (*p* = 0.024), impulsivity (*p* = 0.031), and inattention (p = 0.001) also demonstrated significant differences between the groups. However, the adjusted *p*‐value for multiple comparisons correction was significant for the Conners’ total score (*p* = 0.008) and inattention (*p* = 0.004).

**TABLE 2:** Outcomes resulting from Conners' Parent Rating Scale‐revised in the intervention and control groups.

Characteristics	Group	Before intervention	After intervention	*p*‐value	Differential
Conners score	Methylphenidate	75.05 ± 14.17	75.05 ± 11.35	1.000	−2 (−19–34)
	Methylphenidate/Saffron	74.41 ± 15.52	61.91 ± 15.26	**<0.001**	−10 (−38–9)
	***p*‐value**	0.889	**0.009**		**0.002**
Hyperactivity	Methylphenidate	19.81 ± 5.70	19.90 ± 4.02	0.926	−1 (−6–12)
	Methylphenidate/Saffron	20.41 ± 6.79	16.09 ± 5.56	**0.011**	−2 (−30–8)
	***p*‐value**	0.756	**0.019**		0.024
Impulsivity	Methylphenidate	22.19 ± 5.21	21.43 ± 4.04	0.423	−1 (−8–14)
	Methylphenidate/Saffron	20.23 ± 5.60	17.04 ± 5.95	**0.012**	−3 (−13–8)
	***p*‐value**	0.241	**0.006**		0.031
Inattention	Methylphenidate	34.76 ± 5.76	34.05 ± 6.20	0.487	−1 (−9–8)
	Methylphenidate/Saffron	35.41 ± 8.36	28.82 ± 8.72	**<0.001**	−6 (−20–4)
	***p*‐value**	0.770	0.058		0.001

Table 3 presents the results of the CPT. While there were no significant differences in impulsivity, inattention, and sustained attention between the groups or over time, the reaction time significantly improved the intervention group from pre‐ to post‐intervention (*p* = 0.023). However, after Bonferroni correction for multiple comparisons (adjusted *p* < 0.0125), only the Conners’ total score (*p* = 0.008) and inattention (*p* = 0.004) remained significant.

**TABLE 3:** Outcomes resulting from continuous performance test in the intervention and control groups.

Characteristics	Group	Before intervention	After intervention	*p*‐value	Differential
Impulsivity	Methylphenidate	6 (1–37)	4 (0–67)	0.885	−2 (−15–64)
	Methylphenidate/saffron	3.5 (0–70)	2.5 (0–44)	0.365	−1.5 (−69–43)
	***p*‐value**	0.654	0.549		0.814
Inattention	Methylphenidate	4 (0–25)	3 (0–9)	0.168	0 (‐22–8)
	Methylphenidate/saffron	3.5 (0–28)	2 (0–9)	0.089	−1 (−28–5)
	***p*‐value**	0.994	0.474		0.336
Sustain attention	Methylphenidate	135.05 ± 11.80	136.33 ± 18.29	0.783	3 (−72–26)
	Methylphenidate/saffron	128.23 ± 27.04	139.32 ± 13.29	0.097	2 (−48–100)
	***p*‐value**	0.294	0.407		0.961
Reaction time	Methylphenidate	616.09 ± 111.71	603.05 ± 85.10	0.490	−16 (−159–269)
	Methylphenidate/saffron	612.32 ± 84.84	579.36 ± 86.74	**0.023**	−35 (−163–107)
	***p*‐value**	0.901	0.337		0.437

Table 4 displays the results from the CBST. Working memory span and working memory scores were comparable between the groups at various time points throughout the study. Moreover, there were no significant changes in scores within either group during the study, and the trends over time did not differ significantly between the two groups.

**TABLE 4:** Outcomes resulting from Corsi block‐span task in the intervention and control groups.

Characteristics	Group	Before intervention	After intervention	*p*‐value	Differential
Working memory span	Methylphenidate	3.00 ± 1.84	3.19 ± 1.89	0.568	0 (−4–3)
	Methylphenidate/saffron	3.09 ± 1.95	3.23 ± 1.87	0.678	0 (−3–5)
	***p*‐value**	0.876	0.941		0.628
Working memory score	Methylphenidate	6 (1–48)	6 (1–60)	0.341	0 (−19–34)
	Methylphenidate/saffron	9 (0–48)	19 (0–54)	0.381	0 (−24–29)
	***p*‐value**	0.951	0.863		0.906

Throughout the trial, follow‐up assessments did not reveal any instances of allergy, intolerance, or severe adverse effects related to the prescribed dosages.

## 4 Discussion

Saffron has been reported as an effective adjuvant therapy for several psychiatric disorders (Han et al. 2024; Matraszek‐Gawron, Chwil, Terlecki, and Skoczylas 2022). However, there is limited evidence regarding the efficacy of saffron extract when combined with methylphenidate for managing ADHD in children. This study aimed to evaluate the improvement in ADHD symptoms in children aged 6–12 years through both subjective and objective assessments after six weeks of treatment with saffron extract alongside methylphenidate. Our findings indicated a significant improvement in subjective assessment of hyperactivity, impulsivity, and inattention reported by parents. Additionally, enhancements were observed in working memory and sustained attention (objective assessments) following treatment, but the changes were not statistically significant.

Our results demonstrated that children in the methylphenidate/saffron group showed statistically significant reductions in total ADHD scores and inattention after the intervention compared to the control group receiving only methylphenidate. The Conners' Parent Rating Scale results highlight the potential effectiveness of the combination treatment, suggesting that saffron may enhance the therapeutic effects of traditional pharmacological treatments. This finding aligns with previous studies indicating that saffron may exhibit neuroprotective properties and influence dopaminergic and serotonergic systems, potentially improving mood and cognitive functions (Bian et al. 2020). A double‐blind trial employed 20–30 mg/d saffron capsules depending on weight for six weeks compared to methylphenidate in children 6–17. This study, which assessed subjects using ADHD‐RS‐IV at baseline, weeks 3 and 6, showed similar efficacy for saffron and methylphenidate in children (Baziar et al. 2019). Similar to our trial, this study lacked a placebo group, which complicates attributing improvements solely to saffron versus non‐specific effects. Similarly, a non‐randomized trial on ADHD children aged 7–17 years indicated that the efficacy of saffron after three months is comparable to that of methylphenidate (Blasco‐Fontecilla et al. 2022). Another trial considered adding saffron to methylphenidate for treating children and adolescents with ADHD aged 6–16. After eight weeks, this study revealed better improvement from combining saffron with methylphenidate than alone (Khaksarian et al. 2021). Prescribing saffron adjuvant therapy for adults with ADHD for six weeks also presented improved self‐report symptoms (Pazoki et al. 2022). Although the outcome assessment methods, type of saffron, and the treatment duration were different between studies, outcomes highlight the positive effectiveness of saffron as an alternative or at least adjuvant therapy for ADHD.

A key discrepancy in our findings is the significant improvements in subjective parent‐reported symptoms (via CPRS‐R) contrasted with the lack of significant changes in objective measures of impulsivity, inattention, and sustained attention on the CPT, as well as working memory on the CBST. This may stem from parental expectancy effects in this open‐label design, where knowledge of the adjunctive saffron could influence perceptions of symptom improvement. Alternatively, the objective tasks may not be sensitive enough to detect subtle changes over six weeks, or the effects of saffron may be more pronounced in real‐world behaviors observed by parents rather than controlled lab settings. Methylphenidate's peak effects during school hours also suggest that incorporating blinded teacher reports in future studies could provide a more comprehensive assessment. These contradictory results underscore the need for cautious interpretation, as the observed subjective benefits may not solely reflect saffron's pharmacological action but could include placebo or bias elements.

While our findings indicated no significant differences in impulsivity, inattention, or sustained attention variables measured by the CPT, a noteworthy improvement in reaction time was recorded in the intervention group. This suggests that methylphenidate and saffron treatment may enhance the speed of processing information even if the overarching attention measures did not exhibit the same level of improvement. Although these results were not statistically significant across all domains, they provide preliminary insights into the interplay between cognitive processing speed and ADHD symptomatology, warranting further investigation. However, a study by Blasco‐Fontecilla et al. revealed that saffron improved sustained attention (Blasco‐Fontecilla et al. 2022). Findings concerning the effect of methylphenidate on sustained attention are controversial. In line with the present study, some reports indicated a lack of improved sustained attention following methylphenidate consumption (Merrill et al. 2021). On the other hand, another study revealed a positive change in sustained attention in children with ADHD under methylphenidate treatment (Mizuno et al. 2022). Moreover, reaction time in ADHD children receiving methylphenidate is comparable to children without ADHD (Güven et al. 2019). However, these studies did not investigate the alternative effects of complementary medicines on this function. The lack of difference between the groups in objective‐based items can be attributed to factors such as the complementary medicine dose and the follow‐up time, as well as an acceptable performance in the baseline. Since the dose of the complementary medicine's active ingredient is low, and the patients examined in this study showed severe disease, it may be necessary to change the dosage and increase the duration of use to observe stable and real effects.

Various research has shown that working memory activity increases due to using methylphenidate (Kobel et al. 2009). Also, this effect has been observed in spatial working memory tasks, and fMRI studies have emphasized it (Kobel et al. 2009; Mehta et al. 2000). Moreover, it has been suggested that stimulant medication‐induced changes in spatial working memory are more pronounced in children with a lower baseline of optimal performance (Mehta et al. 2000). However, CBST results indicated no significant differences in working memory capacity in either groups. The lack of change within and between the groups could reflect the complex nature of working memory related to ADHD and the varying impacts of pharmacological and non‐pharmacological interventions. Future research could explore the optimal dosages and treatment durations that may lead to more definitive results in working memory enhancements.

Importantly, our study noted that follow‐up assessments did not reveal any allergic reactions, intolerances, or severe adverse effects associated with the prescribed dosages of saffron, which speaks to its safety profile. This is particularly relevant in pediatric populations, where concerns about the side effects of medications are paramount. The favorable safety profile of saffron may encourage its acceptance among parents and clinicians as an adjunctive treatment option for ADHD.

The present study represents the first clinical trial conducted in Iran to assess the additive effects of saffron on executive function, specifically focusing on working memory in children with ADHD aged 6 to 12 years. While the subjective evaluation of ADHD symptoms was obtained through parent reports, this study also employed objective measures to investigate working memory and sustained attention, utilizing the CPT and CBST. It is important to note that the present study has been focused on children aged 6 to 12 to control the potential impact of age distribution on treatment outcomes.

Several limitations of this study should be acknowledged. Notably, the absence of a placebo in the control group may introduce bias in the parent‐reported outcomes, as parental perceptions can be inherently subjective. This open‐label design limits our ability to attribute improvements specifically to saffron rather than expectancy effects. Additionally, conducted in Iran, where saffron is culturally familiar, results may not generalize to Western countries with different perceptions of herbal medicine, potentially affecting parental reports. Furthermore, although previous studies investigating saffron for ADHD treatment have not reported severe adverse effects, the follow‐up period in this trial may have been too short to fully assess long‐term safety and efficacy; thus, extending the follow‐up duration could yield valuable insights. To address these limitations, we recommend that future randomized placebo‐controlled trials rigorously evaluate treatment effects while incorporating objective behavioral assessments. Additionally, studies should aim for larger and more diverse sample sizes to enhance the generalizability and validity of the findings. To further strengthen future studies, incorporating teacher ratings to capture school‐hour effects and extending follow‐up to 12 weeks or more would provide a more comprehensive understanding of saffron's adjunctive role.

## 5 Conclusion

In conclusion, the results of this study suggest that the combination of saffron and methylphenidate may provide significant improvements in subjective ADHD symptoms, particularly in reducing inattention. While further research is needed to elucidate the mechanisms underlying these effects and determine the long‐term efficacy and safety of saffron, our findings support its potential role as a beneficial adjunctive therapy, but objective measures indicate limited cognitive enhancements. As interest in alternative treatments continues to grow, our study adds to the conversation on how natural supplements can complement traditional pharmacological interventions to enhance outcomes for children with ADHD.

## Author Contributions

**Asieh Hosseinpourfardi**: conceptualization, methodology, visualization, writing – review and editing, writing – original draft, data curation. **Fahimeh Afzaljavan**: methodology, writing – original draft, writing – review and editing, formal analysis, data curation. **Fatemeh Moharreri**: conceptualization, investigation, methodology, funding acquisition, writing – review and editing, supervision, resources, project administration. **Atefeh Soltanifar**: conceptualization, writing – review and editing, supervision, resources. **Seyyed Saeid Sahaf**: methodology, visualization, writing – review and editing, formal analysis, data curation.

## Funding

This study was financially supported by Mashhad University of Medical Sciences under the grant number 4020058.

## Ethics Statement

The study protocol received approval from the Ethics Committee of Mashhad University of Medical Sciences (Ethics Number: 1402.386). The study is registered in the Iranian Clinical Trial Registration Center (Registration Number: IRCT 1130005280N63). Prior to participation, parents (either father or mother) were thoroughly informed of the intervention procedures, potential side effects, and benefits, and they provided written informed consent.

## Data Availability

The data that support the findings of this study are available from the corresponding author upon reasonable request.
